# Editorial: Impact of system biology and molecular medicine on the management of complex immune mediated respiratory diseases, volume II

**DOI:** 10.3389/fmed.2023.1187941

**Published:** 2023-06-07

**Authors:** Blanca Cárdaba, Girolamo Pelaia

**Affiliations:** ^1^Immunology Department, IIS-Fundación Jiménez Díaz, Universidad Autónoma de Madrid, Madrid, Spain; ^2^Center for Biomedical Network of Respiratory Diseases (CIBERES), ISCIII, Madrid, Spain; ^3^Department of Health Sciences, University “Magna Græcia” of Catanzaro, Catanzaro, Italy

**Keywords:** asthma, chronic obstructive pulmonary disease, respiratory inflammation, biological therapies asthma, COVID, metabolomic, miRNA

Complex Immune-Mediated Respiratory Diseases are highly complex and heterogeneous inflammatory disorders, sharing a common organ-disease target which is the lung, but including a broad clinical spectrum. The most frequent obstructive respiratory diseases include bronchial asthma and, chronic obstructive pulmonary disease (COPD). These disorders are characterized by airflow limitation, cough, dyspnea, chest tightness, shortness of breath, and mucus production that could be caused by numerous environmental agents, as well as, genetic, pharmacologic, physiologic, biological, or immunologic mechanisms, which give rise to distinct phenotypes, with underlying molecular mechanisms or endotypes that need to be understood. This great heterogeneity translate to a lack of good therapeutic options for an important percentage of patients that do not respond to standard treatments, who could receive relevant benefits by a precision or personalized strategy, which requires new diagnostic and therapeutic approaches. Our purpose in the second volume of this Research Topic was to collect the latest advances in the molecular and clinical characterization of these complex diseases, in order to better understand the underlying mechanisms and improve the overall management.

In this volume, five novelty works have been published: 4 original articles, 2 of them related to severe asthma, and the other 2 regarding COPD. The fifth is a mini review that summarizes the latest new advances about the T cell-mediated lung inflammation in COVID-19 infection.

In regard to bronchial asthma studies, the two published articles pursue a better understanding of underlying mechanisms of severe uncontrolled asthma, thus searching for cellular and molecular pathways involved in the pathobiology of asthmatic endotypes that do not respond to standard asthma treatments and remain uncontrolled, thereby being responsible for exacerbations and hospitalizations every year.

Asthma is a common, chronic respiratory disease, defined as “a heterogeneous disease, usually characterized by chronic airway inflammation” by the Global Initiative of Asthma (1). Despite its clinical heterogeneity, allergic mechanisms have been implicated in 50–80% of asthmatic patients and in ~50% of severe asthma cases (2, 3). Such considerations explain why there are several new biological treatments mainly directed against this subtype of inflammation (type 2 inflammation). Overall, treatment for asthmatic patients is complex and there is not an asthma cure, but in a large majority of patients multiple combinations of inhaled corticosteroids, short- and long-acting bronchodilators, leukotriene modifiers and anticholinergics provide a satisfactory disease control. In more severe asthma phenotypes, new biological therapies can be used with good clinical results. However, there is still a group of patients who do not respond to any treatment and remain uncontrolled, thus experiencing a very low quality of life. Indeed, these patients are both allergic and non-allergic subjects.

In this Research Topic, Delgado Dolset et al. published a work where they analyzed the implication of allergy in the uncontrolled severe asthmatic phenotype. In particular, they used a very interesting metabolomic research strategy, applied to the sera from two kinds of patients with uncontrolled severe asthma, such as uncontrolled house dust mite-allergic asthma (UCA) and uncontrolled, non-allergic asthma (UCNA) patients. Their results show important metabolic differences between UCA and UCNA patients, mainly concerning an increase in a set of lysophosphocholines (LPCs) associated to the phospholipase A2 (PLA2) pathway, together with a decrease in palmitoleylcarnitine, that points to the arachidonic acid (AA) pathway. They conclude that allergy induces the activation of specific inflammatory pathways, including the AA pathway, along with a dysregulation of LPC mediators, which supports its implication in the uncontrolled asthma phenotype. However, in the non-allergic severe asthmatic patients the authors did not find clear therapeutic targets, thereby concluding that it is necessary to carry out more studies in these patients needing more specific therapeutic tools.

The next work analyzes other unsolved questions in asthma, namely why there is a poor response to glucocorticoid (GC) therapy in ~10% of asthmatic patients (4–7). Hu et al. continued to investigate along the track of previous works reporting that GC-induced transcript 1 (GLCCI1) deficiency in a model of asthma mice led to GC resistance by the decrease of glucocorticoid receptor (GR) expression (8). In this further study, the authors extensively explored the function of GLCCI1 expressed by airway epithelial cells in regulating GC responses via the GR-glucocorticoid receptor interacting protein 1 (GRIP1) pathway and in an Ovalbumin (OVA)-induced asthma model. Also was studied the function of interferon regulatory factor (IRF) family members (IRF1 and IRF3), competing with GR for GRIP1 binding under GLCCI1-deficient conditions. Their results, obtained using appropriate experimental approaches (animal model and human cellular assays), were consistent with the idea that the loss of GLCCI1 expression leads to GC insensitivity through down-regulation of the GR-GRIP1 pathway. This change is associated with enhanced interactions between IRF1 and IRF3 with GRIP1 in asthma. Such data could explain, at least in part, the loss of corticosteroid efficacy in severe uncontrolled asthma. Moreover, these authors found that GLCCI1 deficiency upregulated the chemokines CCL4 and CCL7, thus leading to decreased steroid responsiveness. Taken together, these results suggest that the chemokine production induced by the GLCCI1 deficiency can be attributed to the GRIP1 pathway, and in author's opinion this finding might address interesting future research.

In summary, both works, published by Delgado Dolset et al. and Hu et al. through very different experimental approaches, give new molecular insights, potentially useful to improve the management of severe uncontrolled asthma.

Next, two COPD-related works have been included in this Topic. COPD affects 384 million people worldwide and is the third leading cause of death globally (9). Like asthma, COPD is a heterogeneous respiratory syndrome with two main disease phenotypes, i.e., chronic obstructive bronchitis and pulmonary emphysema (10–12), but with different inflammatory endotypes (13) that need to be further understood. Here, Burke et al. explored for the first time the predictive ability of extracellular vesicles (EV) miRNA from the lungs of patients with COPD [by studying broncoalveolar lavage fluid (BALF)], to discriminate mild COPD patients from healthy controls subjects, and evaluated their relationship with inflammatory endotypes. Their work identified five upregulated and three downregulated miRNA in COPD BALF EVs. Also, a correlation of two specific miRNA with inflammatory cell numbers was found in COPD. These findings suggest the diagnostic potential of specific lung derived EV miRNA, even in mild COPD and, pointing to a role in defining inflammatory endotypes, which could be useful for treatments stratification.

Another essential unsolved question in COPD management is to understand and prevent COPD exacerbations, which are the leading causes of hospitalization, and significantly contribute to morbidity and mortality. Here, Guo-Parke et al. address one of the major causes of exacerbations in COPD patients, namely the human rhinovirus (HRV) infection. They analyzed the pathological changes induced in bronchial epithelial cells after HRV infection, by assessing barrier integrity and inflammatory response in bronchial epithelium cells from patients with severe COPD after virus infection, compared with healthy control subjects. The results showed important differences between COPD and healthy controls. Although HRV infection compromised the tight junction integrity in both kinds of cultures, there was heightened disruption in COPD cultures. HRV induced more pathological changes (sloughing, apoptosis and mucus hypersecretion) in epithelial cells from COPD patients than in healthy control epithelial cells. A Th1/Th2 imbalance and strong interferon and pro-inflammatory cytokine responses were also observed in infected-cells from COPD patients. They conclude that the susceptibility from severe COPD patients to respiratory virus-induced exacerbations is mainly due to molecular changes in host airway epithelium and the preexisting inflammatory environment, rather than viral replication per se. They suggest that COPD inflammation and defects in host differentiation profiles may provide potential therapeutic targets for prevention and management of severe COPD exacerbations.

Finally, Shakiba et al. published a review of the role of T-cells resident in the lung of COVID-19 patients, thus focusing on the reported T cell phenotypes in mild, moderate, and severe COVID-19. In particular, these authors assessed lung inflammation, reviewing the implication of T cells in the disease course and protection. They conclude that T cells in the lung have a dual role during COVID-19, with a more coordinated/protective response in mild/moderate disease, and a dysfunctional/tissue-damaging response in severe disease. They also remark the importance of the other immune cell types, especially lung neutrophils and macrophages, thereby disclosing a dysfunctional response in severe COVID-19, which contributed to extensive tissue damage and fibrosis. Finally, they highlighted the need to study more in depth several aspects of T cell after infection or vaccination (i.e., function, activation, T cell monitoring, and dynamics of TCR repertoire of SARS-CoV-2 specific T cells), as well as to better elucidate the role of T cells in the pathophysiology of long-COVID.

In summary, this Research Topic describes important advances in the understanding of the molecular complexity underpinning widespread respiratory diseases such as asthma and COPD. Similar to the first volume, this second one reflects the high complexity of respiratory diseases and the many efforts, sustained from different points of view that are needed to improve the management of such disorders, which affect a high number of subjects around the world. We express many thanks to the authors and researchers who have contributed to this Topic, and we also hope that this issue will help readers to understand the extent to which efforts in this field are needed in order to provide a better quality of life to patients.

## Author contributions

BC and GP wrote and reviewed the manuscript. All authors contributed to the article and approved the submitted version.
